# Great occipital nerve long-acting steroid injections in cluster headache therapy: an observational prospective study

**DOI:** 10.1007/s00415-021-10884-0

**Published:** 2021-11-25

**Authors:** Elena Merli, Gian Maria Asioli, Valentina Favoni, Corrado Zenesini, Davide Mascarella, Alex Sartori, Pietro Cortelli, Sabina Cevoli, Giulia Pierangeli

**Affiliations:** 1 IRCCS Istituto delle Scienze Neurologiche di Bologna, Ospedale Bellaria, via Altura 3, Bologna, Italy; 2 DIBINEM, Department of Biomedical and Neuromotor Sciences, University of Bologna, Bologna, Italy

**Keywords:** Cluster headache, Transitional therapy, Great occipital nerve, Methylprednisolone

## Abstract

**Background:**

Injections targeting the occipital nerve are used to reduce headache attacks and abort cluster bouts in cluster headache patients. There is no widely accepted agreement over the optimal technique of injection, type and doses of steroids and/or anesthetics to use, as well as injection regimens. The aim of this study was to verify the effectiveness and safety of greater occipital nerve long-acting steroid injections in the management of episodic and chronic cluster headache.

**Methods:**

We conducted a prospective observational cohort study on episodic (ECH) and chronic cluster headache patients (CCH). ECH were included in the study at the beginning of a cluster period. Three injections with 60 mg methylprednisolone were performed on alternate days. We registered the frequency and intensity of attacks three days before and 3, 7 and 30 days after the treatment, the latency of cluster relapse, adverse events, scores evaluating anxiety (Zung scale), depression (Beck’s Depression Scale) and quality of life (Disability Assessment Schedule II, 12-Item Self-Administered Version). Primary outcome was the interruption of the cluster after the three injections. Responders conducted a follow-up period of 12 months.

**Results:**

We enrolled 60 patients, 47 with ECH and 13 with CCH. We observed a complete response in 47.8% (22/46) of episodic and 33.3% (4/12) of chronic patients. Moreover, a partial response (reduction of at least 50% of attacks) was obtained in further 10.8% (5/46) of episodic and in 33.3% (4/12) of chronic patients at 1 month. Median pain-free period was of 3 months for CCH responders. Only mild adverse events were reported in 38.3% (23/58) cases.

**Conclusions:**

We suggest three greater occipital nerve injections of 60 mg methylprednisolone on alternate days as useful therapy in episodic and chronic cluster headache. This leads to a long pain-free period in chronic forms. Adverse effects are mild and support its use as first choice.

**Trial registration:**

The study was inserted in AIFA observational studies register.

**Supplementary Information:**

The online version contains supplementary material available at 10.1007/s00415-021-10884-0.

## Introduction

Cluster headache (CH) is among the most painful headache disorders. An attack of severe, unilateral, orbital or temporal pain, along with ipsilateral cranial autonomic features and a sense of restlessness may occur up to 8 times a day and last from 15 to 180 min [1]. In episodic CH (ECH), attacks occur in series lasting for weeks or months (so-called cluster periods) separated by remission periods usually lasting months or years. In 10–15% of patients, CH begins as or evolves into a chronic form (chronic CH, CCH), in which remission periods last less than three months or are completely absent [1]. Medical treatment of CH includes acute, transitional and preventive therapy. Acute treatment aims to abort the pain of each attack, while preventive therapy modulates attack frequency, intensity and cluster duration. Oral preventive treatments, such as verapamil, lithium or valproate, frequently cause side effects and need days or weeks to be active. Transitional therapy should provide almost immediate relief to decrease pain until preventive therapy becomes effective or until the cluster period ends spontaneously [2]. Although transitional therapy commonly consists of oral corticosteroids [3], injections targeting the greater occipital nerve (GON) are a local alternative to reduce headache attacks and abort cluster bouts [4].

Patterns of use of GON injections greatly vary, as there is no widely accepted agreement among headache specialists over the optimal injection regimens, type and dose of steroids and/or anesthetics [5].

The most numerous randomized placebo-controlled trial indicates a protocol of three repeated steroid injections on alternate days [6]. Drawing inspiration from that protocol, we selected methylprednisolone which is a low-cost and easily available medication and whose use is supported by most previous studies on GON injections.

Therefore, we hypothesized that repeated injections of long-acting steroid could be useful in CH treatment. The aim of our study was to verify the effectiveness and safety of GON long-acting steroid injections alone in the management of ECH and CCH patients.

## Materials and methods

This is an observational prospective cohort study. We consecutively recruited all patients with ECH and CCH attending the Headache Center of IRCSS—Istituto delle Scienze Neurologiche di Bologna, from October 2017 to October 2019 and fulfilling inclusion criteria. Inclusion criteria were: subjects over the age of 18; diagnosis of ECH and CCH according to the criteria of “The international classification of headache disorders” ICHD Edition 3-beta [1]; ECH in active cluster within the first week from cluster onset and CCH during the exacerbation phase; if taking preventive therapies, these should have not been modified in the previous 3 months. As ICHD3 diagnostic criteria for cluster headache were published after the study initiation, we maintained the interim ICHD3-beta criteria throughout the study. Exclusion criteria were: patients with another type of headache; patients with non-active cluster headache; patients with contraindications to methylprednisolone; patients taking anticoagulants or with coagulation disorders; patients using oral steroid therapy; patients with preventive therapies introduced or modified in the previous 3 months; patients unable to sign the informed consent. The protocol consisted in three injections on alternate days. Each injection was performed on the side of the pain using a syringe with 60 mg of methylprednisolone solved in 2 ml of saline solution, with a 25-gauge needle. GON was localized by presuming a line from the occipital protuberance to the mastoid process and moving 1/3 of the way laterally as previously reported [7]. The insertion was performed 2 cm below the occipital nuchal ridge, carefully aspirating before injecting, to ensure that the needle was not inside a vessel.

Visits occurred at baseline (preliminary visit T0), 3 days after baseline (T1), 2 and 4 days after T1 (T2 and T3), 1 month after treatment (T4), then at 3 months (T5), 6 months (T6) and 12 months (T7) after the end of treatment.

At T0, while recruiting the patient, inclusion and exclusion criteria were verified and all information regarding participation in the study and treatment of personal data was discussed. A free informed consent form was signed. Previous clinical history was traced (age of onset of CH, duration of previous clusters, characteristics of pain, frequency and intensity of attacks, previous and concomitant preventive therapies, comorbidities, lifestyle). Patients were asked to daily fill in a headache diary, detailing the number of attacks, rating pain severity using the Numerical Rating Scale, and assessing acute therapies used. Diaries were recollected at each follow-up visit. The infiltrations were performed at T1, T2 and T3. One month after the last injection (T4) we asked patients to return their headache diary, and asked patients to complete questionnaires. The complete responders group had also a follow-up visit at 3, 6 and 12 months to verify the relapse of the cluster. At each visit they filled in tests regarding anxiety (Zung’s scale) [8], depression (Beck’s Depression Scale) [9] and quality of life (Disability Assessment Schedule II, 12-Item Self-Administered Version) [10].

### Outcome measures

Primary outcome was to evaluate the number of complete responders, i.e. those with complete disappearance of attacks after the third infiltration and who maintained the efficacy at least for the first month. Secondary outcomes were to evaluate the number of partial responders, i.e., those with an improvement of at least 50% in the frequency of attacks after one month from treatment compared to previous frequency, the reduction of the intensity of pain, the evaluation of recurrence of cluster attacks at one-year of follow-up, the improvement of anxiety, depression and quality of life. We considered a no response as the absence of any significant therapeutic effect.

### Standard protocol approvals and patient consents

The study was approved by the Local Ethics Committee of the health service of Bologna (Comitato Etico Indipendente Area Vasta Emilia Centro: CE 17130). All patients gave their written informed consent to study participation.

### Statistical analysis

Continuous variables were presented as mean and standard deviation (SD) or median and interquartile range (IQR), the normality of distribution was evaluated with Shapiro-Wil test. Categorical variables were presented as absolute (*n*) and relative frequency (%). Analyses were performed for all patients and for ECH and CCH patients sub-groups.

The primary and secondary outcomes were presented as absolute (*n*) and relative frequencies (%). Wilcoxon matched pairs signed-ranks test was used to compare pain intensity and number of attacks over the time at 3, 7 and 30 days after the baseline. Wilcoxon matched pairs signed-ranks test was used to compare Zung’s scale, Beck’s Depression Scale and Disability Assessment Schedule II scores at 30 months compared to the baseline. The results were presented as median reduction and interquartile range reduction compared to the baseline. Finally, we used Kruskal–Wallis test to compare the variations of the scales above and duration of illness between the three groups: responders, partial and not responders. Two-sided *p* values were presented.

Statistical analysis was performed using statistical package Stata SE, 14.2.

## Results

Sixty CH patients were enrolled: 47 ECH and 13 CCH. Demographic and baseline characteristics are included in Table 1. Oral preventive therapy was ongoing in 10 out of 13 CCH patients (76.9%) and in 18 out of 47 ECH patients (38.3%). One ECH discontinued the study after the first injection because of trypanophobia. One CCH was lost at follow-up. Among the remaining 58 patients, 26 (44.8%) were attack free after 1 month from the third injection. Comparing ECH and CCH patients, complete responders were 22/46 (47.8%) and 4/12 (33.3%), respectively (Table 2). Furthermore, 5/46 of ECH (10.8%) and 4/12 of CCH (33.3%) were partial responders reaching the secondary outcome. Hence, altogether, about 2/3 of CCH ameliorated their frequency of attacks/day of > 50% from the baseline. We describe a slight, non-significant improvement in pain intensity throughout the month even in non-responders. Detailed results on the reduction of frequency and intensity of pain during the month are reported on Table 2. Among the analyzed variables, duration of CH since onset negatively correlates with a good response to treatment: responders had a short duration of CH (median 10 years since CH onset (IQR 5–18) compared to non-responders (median 20 years (IQR 11–26); *p* = 0.022). On the contrary, previous response to oral steroid therapy, the frequency, intensity or duration of attacks, sex, smoking, and concomitant therapies did not correlate with treatment response. A proportion of 23 patients of the whole sample (38.3%) reported mild adverse events: 21 complained of neck stiffness and 2 of mild pain on the site of injection. Among them, five patients (8.3%) still complained of neck stiffness after 1 month. No serious nor systemic adverse events were observed. Complete responders underwent a 1-year follow-up: 50% of ECH were still pain-free at 1-year of follow-up, while 50% had recurrence of cluster attacks with a mean latency of 8 months (242 days, SD ± 130.49). All CCH patients relapsed with a median pain-free period of 3 months (94 days, SD ± 7.85). Zung’s scale, Beck’s Depression Scale and Disability Assessment Schedule II at one month revealed a slight improvement compared to baseline. No significant difference was shown comparing responders to non-responders (Table 3).

**Table 1:** Description of CH patients at baseline

	Total CH *N* = 60	CCH *N* = 13	ECH *N* = 47
Sex			
M—*n* (%)	50 (83.3)	11 (84.6)	39 (83.0)
F—*n* (%)	10 (16.7)	2 (15.4)	8 (17.0)
Age—mean (SD)	44 (11.9)	46.1 (15.6)	43.7 (10.8)
Age of onset—median (IQR)	27 (20–31)	34 (25–40)	25 (19–30)
Duration of illness (y)—mean	16.6	11.8	17.9
Smoke			
Non-smoker—*n* (%)	14 (23.3)	1 (7.7)	13 (27.6)
Previous smoker—*n* (%)	18 (30)	4 (30.8)	14 (29.8)
Active smoker—*n* (%)	28 (46.7)	8 (61.5)	20 (42.6)
Number of daily attacks—median (IQR)	2 (1–3)	2 (1–3)	2 (1–3)
Intensity of attacks—median (IQR)	8 (7–9.5)	8 (8–9)	8 (7–10)
Duration of CH since onset—median (IQR)	16 (7–23)	11 (5–18)	17 (10–26)
Frequency of clusters/year—median (IQR)	–	–	1 (0.5–1)
Duration of cluster (month)—median (IQR)	–	8 (5–9)	1.5 (1–2)
For CCH only			
Chronic at onset—*n* (%)		6 (46.2)	
Episodic at onset—*n* (%)		7 (53.8)	
Ongoing preventive therapy			
Yes—*n* (%)	28 (46.6)	11 (84.6)	17 (36.1)
No—*n* (%)	32 (53.3)	2 (15.4)	30 (63.83)
Type of preventive therapies			
- Verapamil		6	17
- lithium		1	–
- Verapamil plus lithium		2	1
- Verapamil plus valproate		1	–
Previous oral steroid therapy—*n* (%)	14 (23.3)	1 (7.7)	13 (27.6)
Response Yes—*n* (%)	8 (57.1)	1 (100)	7 (53.8)
Zung’s Scale score at T0—mean (SD)	33.34 (5.70)	33.54 (6.28)	33.28 (5.61)
Beck’s Depression Scale score at T0—mean (SD)	10.74 (8.06)	10.62 (8.36)	10.77 (8.07)
WHODAS 2.0 score at T0—mean (SD)	17.76 (14.30)	14.47 (12.65)	18.54 (14.70)

*CH* cluster headache, *ECH* episodic cluster headache, *CCH* chronic cluster headache, *SD* standard deviation, *IQR* interquartile range, *y* year

**Table 2:** Results

	Total CH (*N* = 58) *n*—%	CCH (*N* = 12) *n*—%	ECH (*N* = 46) *n*—%
Complete responders^a^	26 (44.8)	4 (33.3)	22 (47.8)
Partial responders^b^	9 (15.5)	4 (33.3)	5 (10.8)
Median reduction of n of attacks/day			
T2	− 0.6	− 2	− 0.55
T3	− 0.55	− 0.5	− 0.55
T4	− 0.8	0	− 0.1

*CH* cluster headache, *ECH* episodic cluster headache, *CCH* chronic cluster headache, *SD* standard deviation; ^§^Complete responders = those with complete disappearance of attacks after the third infiltration and who maintained the efficacy at least for the first month; ^†^Partial responders = improvement of at least 50% in the frequency of attacks after one month from treatment compared to previous frequency

**Table 3:** Anxiety, depression and quality of life evaluation at Day 30 compared to Day 0

Mean reduction - mean (SD)	Zung’s scale	Beck’s Depression Scale	Disability assessment schedule II
Complete responders^a^	− 0.23 (4.7)	− 5.25 (8.34)	− 9.58 (14.7)
Partial responders^b^	− 1. 25 (5.0)	− 2.87 (2.41)	− 7.4 (7.7)
Non-responders	− 3 (4.5)	− 2.6 (5.9)	− 1.0 (11.6)
*P*	0.285	0.476	0.442

*CH* cluster headache, *ECH* episodic cluster headache, *CCH* chronic cluster headache, *SD* standard deviation; ^a^Complete responders = those with complete disappearance of attacks after the third infiltration and who maintained the efficacy at least for the first month; ^b^Partial responders = improvement of at least 50% in the frequency of attacks after one month from treatment compared to previous frequency

## Discussion

Our study shows the effectiveness and safety of GON long-acting steroid injections alone as a transitional therapy in a large sample of CH patients. This is the first study that evaluated the use of methylprednisolone at the high dosage of 60 mg, repeated in three injections on alternate days on the side of pain. We found a high efficacy in stopping the cluster period: 45% of patients (48% of ECH and 33% of CCH) were attack free for at least one month after the third injection. Moreover, a further proportion of 11% of ECH and 33% of CCH diminished the frequency of attacks per day of at least 50%, reaching the secondary outcome.

A variety of studies suggest that GON injections represent a safe and useful therapy in CH (see Table 4) [2, 5, 6, 11–18]. In spite of this, data from literature are not easily comparable due to the differences in inclusion criteria, protocol of treatment, type and doses of steroid and/or anesthetics used and clinical outcome observed. Most protocols provide a combination of steroid plus anesthetic injection or a simple anesthetic block, however, only a few studies can boast an adequate randomized, placebo-controlled design. In the most numerous one [6], Leroux proved the efficacy of three repeated injections on alternate days of cortivazol in ECH and CCH. Drawing inspiration from the experience of Leroux, we selected methylprednisolone which is a low-cost and easily available medication and whose use is supported by most previous studies on GON injections. Overall, in previous studies a complete response was variably shown in 28–63% of CH (25–63% in ECH; 30–90% in CCH) (see Table 4). Our results are in line with the ones reported.

**Table 4:** Review of the literature

Study	Patients	Drug	No. inj	Outcome	AE (%)	Type of Study
Bigo et al. [11]	8 ECH + 8 CCH	Methylprednisolone 160 mg	Single	ECH: CR 25%; PR 12.5% (not better defined); NR 50%	15.9	Observational retrospective
				CCH: PR 50%; NR 50%		
Peres et al. [2]	9 ECH + 5 CCH	Lidocaine 1% 3 mL and triamcinolone 40 mg	Single	Total CH: CR 28.5% (pain-free period lasting longer than 2 weeks); PR 35.7% (pain-free period of less than 2 weeks); NR 35.7%	NA	Observational retrospective
				Mean duration of pain freedom in CCH CR: 13.1 days		
Afridi et al. [12]	19 CH	Lidocaine 2% 3 mL and methylprednisolone 80 mg	Single	Total CH: CR 53%; PR 16% (not better defined); NR 31%	NA	Observational retrospective
				Mean duration of pain freedom in CCH CR: 17.2 days		
Gantenbein et al. [13]	31 ECH + 29 CCH	Betamethasone 21 mg and lignocaine 2% 2 mL	Single	ECH: CR 63%; PR 24% (> 25% benefit in intensity or frequency); NR 13%	14.2	Observational retrospective
				CCH: CR 30%; PR 43%; NR 27%		
				Mean duration of pain freedom in CCH CR: 17.2 days		
Gaul et al. [14]	101 CH	Triamcinolone 10 mg and bupivacaine	Single	ECH: CR 62.7% (pain-free period duration not defined); PR 19.7% (not better defined); NR 19.5%	10.9	Observational prospective
				CCH: CR 50%; PR 27.5%; NR 22.5%		
				Mean duration of pain freedom in CCH CR: 14.3 days		
Gönen et al. [15]	51 CH	Betamethasone dipropionate 12.86 mg and betamethasone sodium phosphate 5.26 mg and lidocaine 0.5 ml	Single	Total CH: CR 54.9%; PR 41.18% (not better defined); NR 3.92%	9.8	Observational prospective
Lambru et al. [16]	83 CCH	Lidocaine 2% 2 mL and methylprednisolone 80 mg	Single	CCH: CR 42% [7-day pain-free period]; PR 15% (decrease ≥ 50% in pain intensity/frequency during at least 7 days); NR 43%	82	Observational prospective
				Mean duration of pain freedom in CCH CR: 21 days		
Rozen et al. [17]	10 CCH	Lidocaine 1% 9 mL and triamcinolone 1 mL 40 mg	Single	CCH: CR 90%; PR10%; NR 0%	10	Observational retrospective
				Mean duration of pain freedom in CCH CR: 65 days		
Ambrosini et al. [18]	23 CH	Betamethasone dipropionate 12.46 mg and betamethasone disodium phosphate 5.26 mg and lidocaine 2% 0.5 mL vs physiological saline and lidocaine 2% 0.5 mL	Single	Total CH treated: CR 85% (7 days free of pain); PR NA; NR NA vs Total CH not treated: 0% CR	NA	Randomized placebo-controlled
				Mean duration of pain freedom total CH treated: 41.7 days		
Leroux et al. [6]	18 ECH + 15 CCH	Cortivazol 3.75 mg vs physiological saline	Repeated	Total CH treated: 95% PR (with a mean of two or fewer attacks per day in the second, third, and fourth day after the third injection) vs total CH not treated: 55% PR	21	Randomized placebo-controlled

*inj* injections, *AE* adverse events, *CH* cluster headache, *ECH* episodic cluster headache, *CCH* chronic cluster headache, *CR* complete response, *PR* partial response, *NR* no response, *NA* no available data

Since comparable results were achieved in protocols with local anesthetic or steroids alone, we may suppose that a combination of anesthetics plus steroids does not produce a significantly better response. The clinical benefit is therefore most likely attributable to the steroid. Interestingly, our study demonstrates for the first time that previous response to oral steroid therapy does not correlate with that of steroid injection therapy. This supports the idea of a prevalent local mechanism of action of GON injection, which is not linked to the systemic action of the steroids. In fact, GON is traditionally thought to be a therapeutic target due to the anatomo-physiological convergence of C2 dermatome and trigeminovascular afferents in the spinal trigeminal nucleus, which underlie the reported pain from orbitofrontal regions [19].

The long follow-up is certainly a strong point of our study. After one-year from injection 50.0% of ECH complete responders were still cluster free while the other half relapsed with a mean latency of 8 months. A relevant result is that CCH complete responders obtained a median pain-free period of 3 months, substantially longer than previously reported (13–65 days). This relatively long pain-free period leads us to hypothesize that this repeated protocol over time could be particularly useful in a subset of patients with drug-resistant CCH. Further ad hoc research is needed to confirm this hypothesis.

Furthermore, we confirm the low frequency and severity of adverse events. This certainly supports the choice of local against oral steroid therapy, especially when other cardiovascular or gastric comorbidities are present.

The absence of a control group is a limit of our study, as clusters may change following their natural course and independently of therapeutic approaches. However, in our sample the short duration of CH since onset positively correlates with a good response to GON blockade. Thus, a significant impact of cluster early termination on rate of response is unconvincing, though not totally excludable.

The observational design of our study obviously makes it susceptible to placebo effect, which has also been described in CH patients [20]. However, the long persistence of the response makes it unlikely. A clear point of strength is the consecutive recruitment of patients, which grants the representativeness of the group studied for the larger population of patients followed at our Center.

## Conclusions

Repeated greater occipital nerve injections of 60 mg methylprednisolone, performed on alternate days, is an effective therapy in CH. We observed a complete response in near half of ECH and in a third of CCH for at least of 1 month after the last injection. This protocol seems particularly useful in the treatment of CCH, leading to a 3-month pain-free period in responder patients. Adverse effects are mild and support its use as first choice compared to oral therapies. More studies are needed to compare these results to other types, dosages and timing of local therapies.

## Supplementary Information

Below is the link to the electronic supplementary material.

Supplementary file1 (XLSX 38 KB)

## Data Availability

All data generated or analysed during this study are included in this published article and its supplementary information files.

## Abbreviations

CH: Cluster headache
ECH: Episodic cluster headache
CCH: Chronic cluster headache
GON: Great occipital nerve
CGRP: Calcitonin gene-related peptide
